# Epidemiological study of dry eye disease in Gansu Province based on mathematical modeling: analysis of regional population characteristics and identification of risk factors

**DOI:** 10.3389/fpubh.2026.1890146

**Published:** 2026-09-24

**Authors:** Lei Jiang, Nan Su, Xingpan Du, Yanjun Yong, Juanping Zhou, Jingyan Zhang, Wei Liang, Yuqiong Mao, Kan Yang

**Affiliations:** 1 Department of Ophthalmology, The First People’s Hospital of Lanzhou, Lanzhou, Gansu, China; 2 Department of Ophthalmology, Gansu University of Chinese Medicine, Lanzhou, Gansu, China

**Keywords:** dry eye disease, Gansu, logistic regression model, random forest model, risk factors

## Abstract

**Objective:**

To investigate the epidemiological characteristics of dry eye disease (DED) in Gansu Province, examine the associations between regional population features and DED, and identify risk factors contributing to disease onset, thereby providing scientific evidence for clinical prevention, diagnosis, and intervention in this region.

**Methods:**

A cross-sectional study was conducted from August 2024 to August 2025 using stratified, multi-stage cluster random sampling. A *post hoc* design-effect analysis was performed to quantify the influence of clustering on prevalence precision, and a mixed-effects logistic regression model was used as a sensitivity analysis to account for the hierarchical sampling structure. A total of 2,000 permanent residents aged 18 years or older were recruited from five geographic regions of Gansu Province. All participants completed a standardized questionnaire and ophthalmic examinations including tear film break-up time (TFBUT), corneal fluorescein staining (CFS), and the Schirmer I test (SIT). Factors associated with DED were evaluated using conventional multivariable logistic regression, mixed-effects logistic regression, and random forest analysis.

**Results:**

Climate type, elevation, sex, daily screen time, sleep quality, and smoking were associated with DED in the conventional multivariable model (all *p* < 0.05). The mixed-effects sensitivity analysis yielded generally, but not entirely, consistent findings after accounting for clustering. Based on mean decrease in accuracy, the random forest model ranked climate type as the most influential predictor, followed by daily screen time and sleep quality. The performance of the random forest model was favorable, with an area under the receiver operating characteristic curve (AUC) of 0.832.

**Conclusion:**

The prevalence of DED in Gansu Province is affected by multiple factors related to regional environment, population characteristics, and lifestyle behaviors. Climate type, daily screen time and sleep quality are key risk factors. Public health interventions should prioritize high-risk regions and high-risk populations.

## 1 Introduction

Dry eye disease (DED) is a global public health issue, not merely ocular discomfort, but a multifactorial chronic syndrome of ocular surface homeostasis imbalance (1, 2). Its core pathophysiology involves TFU dysfunction, manifesting as tear film instability, hyperosmolarity, inflammation, damage, and neurosensory abnormalities, which are interconnected processes (3). Epidemiological estimates indicate hundreds of millions worldwide have DED, with prevalence rising with age and higher in females (4, 5). Modern lifestyle changes, particularly VDT use, aging, pollution, and climate change, drive rising DED prevalence, threatening vision, work, mental health, and quality of life, and imposing socioeconomic burdens (6–8).

China’s vast territory is characterized by diverse geographical and climatic conditions, resulting in marked regional heterogeneity in DED epidemiology. Evidence indicates that DED prevalence is higher in northern regions with arid, windy, and high ultraviolet conditions compared with the warmer and more humid areas (9, 10). Environmental stressors may affect ocular surface homeostasis by increasing tear evaporation, promoting tear film instability, and inducing inflammatory responses (11). Long-term exposure to low-humidity environments can directly accelerate tear evaporation, leading to evaporative-type DED (12).

Gansu Province, located at the junction of the Qinghai-Tibet Plateau, the Loess Plateau, and the Inner Mongolia Plateau, is geographically complex and climatically diverse, making it highly representative at the national level. From northwest to southeast, the region presents markedly different ecological landscapes: the Hexi Corridor as an extremely arid zone, the Loess Plateau in Central Gansu as a semi-arid region, Southeastern Gansu as a temperate semi-humid area, Gannan as a cold and humid highland, and Longnan as a subtropical humid zone. This broad environmental gradient, combined with uneven regional economic development and multiethnic population clusters with diverse lifestyles and work patterns, provides a natural “laboratory” for exploring the complex relationships among geography, climate, population, and disease. Currently, it remains unclear how DED risk varies among residents across these highly heterogeneous regions of Gansu, and how the dominant risk factors evolve geographically.

Beyond environmental exposure, individual genetic susceptibility, lifestyle habits, and systemic diseases collectively form a complex etiological network for DED (13). Advanced age and female sex are classical intrinsic risk factors (4, 14), while extrinsic factors such as prolonged screen use causing abnormal blinking, contact lens wear, and use of certain medications are increasingly recognized contributors (15, 16). In Gansu with unique environmental baseline conditions, how these traditional and emerging risk factors interact with environmental factors, and how their relative weights are distributed, remains a critical question. Traditional univariate analysis or simple multivariable logistic regression models can identify independent risk factors but often struggle to handle complex nonlinear relationships and higher-order interactions among variables.

In this context, integrating conventional epidemiological methods with machine learning approaches may provide additional insights into the complex determinants of DED. Multivariable logistic regression enables estimation of independent associations, whereas random forest analysis can evaluate variable importance and identify potential nonlinear relationships among multiple factors. Combining these complementary approaches may facilitate a more comprehensive understanding of the contributions of environmental and behavioral factors to DED.

Based on these research gaps and methodological needs, this study aims to conduct a multicenter cross-sectional survey to characterize the prevalence of DED among adults across different geographical and climatic zones of Gansu Province, and to reveal its spatial distribution patterns. The study systematically analyzes associations between DED and various factors, including sociodemographic characteristics, behavioral lifestyles, and environmental exposures. By innovatively integrating traditional multivariable logistic regression with random forest machine learning, the study seeks not only to identify independent risk factors but also to elucidate their relative contributions in a comprehensive manner.

## 2 Methods

### 2.1 Participants

A cross-sectional study design was employed. Based on previous studies (17, 18), the anticipated prevalence of DED in this study was approximately 30%, with a two-sided significance level (*α*) set of 0.05 and a permissible absolute error (*d*) of 0.02. The initial minimum sample size was estimated using the conventional formula for a population proportion, $n=Z_{1-\alpha /2}^{2}\times P(1-P)/d^{2}$ , yielding approximately 2,017 participants under the assumption of simple random sampling. Because the study adopted a stratified, four-stage cluster random sampling design, the effect of clustering on the required and effective sample sizes was subsequently evaluated using DEFF = 1 + (*m* − 1)*ρ*, where m denotes the average cluster size and ρ denotes the intraclass correlation coefficient (ICC). The effective sample size was calculated as *n*_eff_ = *n*/DEFF. In the first stage, Gansu Province was stratified into five regions according to natural geography and climatic characteristics: the arid Hexi Corridor, the Longzhong Loess Plateau, the Longdong Loess Plateau, the warm and humid mountainous region of Longnan, and the cold and humid plateau region of Gannan. In the second stage, two counties or districts were randomly selected from each region, resulting in a total of 10 counties/districts. In the third stage, each selected county/district was stratified according to urban–rural status; two urban subdistricts and two rural townships were randomly selected from each county/district, resulting in 40 subdistrict/township clusters. Residents were recruited from the selected communities or villages without attempting to survey all residents. Of the 2,764 residents who completed both the questionnaire and ophthalmic examinations, 2,000 met the eligibility criteria and were included in the final analysis, whereas 764 were excluded.

Inclusion criteria were: (1) Residing in Gansu Province for ≥5 years; (2) Age ≥18 years; and (3) Voluntary participation and provision of written informed consent. Exclusion criteria: (1) Presence of other ocular organic diseases, such as glaucoma, cataract, or keratitis; (2) History of ocular surgery or use of immunosuppressive agents within the past 3 months; or (3) Cognitive impairment or inability to cooperate with questionnaire completion or ophthalmic examinations.

### 2.2 Diagnostic criteria and examination procedures

All participants underwent a standardized ophthalmic examination and questionnaire survey. Ophthalmic examinations were performed by a team of ophthalmologists who received uniform training to ensure consistency in procedures and interpretation. The examination procedures strictly followed the internationally recognized 2017 Tear Film and Ocular Surface Society Dry Eye Workshop II (DEWS II) diagnostic criteria (19, 20).

Specifically, the assessments included: (1) tear film break-up time (TFBUT): Evaluates tear film stability. Interpretation: ≤5 s (severe instability), 5–10 s (mild instability), >10 s (normal). (2) corneal fluorescein staining (CFS): Evaluates ocular surface epithelial damage using the Oxford grading scale; a score ≥1 was considered positive. (3) Schirmer I test (SIT) without topical anesthesia: Measures tear secretion. Interpretation: <5 mm/5 min (severe DED), 5–10 mm/5 min (mild DED), >10 mm/5 min (normal).

Participants were classified as having dry eye disease when they reported one or more dry-eye-related symptoms (including dryness, foreign body sensation, burning, eye fatigue, or fluctuating vision) and exhibited at least one abnormal objective ocular surface test result, defined as TFBUT ≤10 s, Schirmer I test ≤10 mm/5 min, or positive corneal fluorescein staining.

### 2.3 Data collection

Elevation data for participants’ residences were obtained from the Gansu Province Geographic Information Public Service Platform. Based on the Climate Zoning of China, residential locations were classified into four climate types: arid, semi-arid, semi-humid, and humid, which represent the major climatic gradients across Gansu Province. DED status was recorded through a combination of questionnaire survey and medical record review.

Structured face-to-face interviews were conducted to collect participants’ age, sex (male, female), work type (indoor, outdoor), sleep quality, smoking status, alcohol consumption, and daily screen time. Sleep quality was assessed using the Pittsburgh Sleep Quality Index (PSQI) (21); a PSQI ≥8 was classified as poor sleep quality, <8 as good sleep quality. Smoking was defined as ≥1 cigarette/day for ≥1 year; otherwise, participants were classified as non-smokers. Alcohol consumption was defined as drinking ≥1 time/week for ≥1 year; otherwise, participants were classified as non-drinkers. Daily screen time was categorized as <6 h or ≥6 h.

All data were double-entered independently using EpiData version 3.1 and underwent logical verification to ensure accuracy.

### 2.4 Variable definition

The primary outcome of this study was the occurrence of DED. Predictor variables included region, climate type, elevation, sex, age, work type, sleep quality, smoking status, alcohol consumption, eyeglass use, and daily screen time. All categorical variables were dichotomized and numerically encoded to facilitate processing by machine learning algorithms (specific coding scheme is presented in Table 1).

**Table 1:** Coding scheme for categorical variables.

Variable	Category
Regions	Longnan (0); Gannan (1); Southeastern Gansu (2); Central Gansu (3); Hexi corridor (4)
Climate type	humid (0); semi-humid (1); semi-arid (2); arid (3)
Elevation	<2,000 m (0); 2,000–3,000 m (1); ≥3,000 m (2)
Age	18–35 years (0); 36–55 years (1); >55 years (2)
Sex	Male (0); Female (1)
Work type	Indoor work (0); Outdoor work (1)
Sleep quality	PSQI <8 (0); PSQI ≥8 (1)
Eyeglass use	Without eyeglasses (0); with eyeglasses (1)
Daily screen time	≤6 h (0); >6 h (1)
Smoking status	No (0); Yes (1)
Alcohol consumption	No (0); Yes (1)

Before model construction, variance inflation factors (VIF) were calculated for all predictor variables. A VIF >5 indicates severe multicollinearity. The maximum VIFs were 7.027 for Region, 6.884 for climate type, and 6.315 for elevation, whereas the corresponding values for all other predictors were below 2.20. Although none exceeded 10, the three geographic variables exceeded the cautionary threshold of 5, indicating meaningful information overlap. Because Region represented a composite geographic classification incorporating climatic and elevational characteristics, it was not included simultaneously with climate type and elevation in the final conventional or mixed-effects model.

### 2.5 Random forest model construction

Random forest models were constructed using the “randomForest” package in R version 4.5.1. Prior to model development, the dataset was randomly divided into a training set (70%) and a testing set (30%) using a hold-out validation strategy. The training set was used for model construction, whereas the testing set was reserved for independent performance evaluation. A variable-level audit identified no missing values in the DED outcome or any candidate predictor included in the analysis (0/2,000, 0.0% for each variable). Therefore, the approximate median imputation option was not invoked, and all 2,000 participants were retained in the random forest analysi. The number of decision trees (ntree) was set to 500 to ensure stable predictions. For each node split, the number of variables randomly selected (mtry) was set to the square root of the total number of predictors, which is the default setting for classification tasks. The minimum size of terminal nodes (nodesize) was left at the default. Variable importance was assessed using the mean decrease in accuracy, with higher values indicating greater contribution to model performance. The number of decision trees was set to 500 to ensure model stability. For each node split, the number of variables randomly selected was set to the square root of the total number of variables, which is the default optimized value for classification tasks. Variable importance was assessed by calculating the mean decrease in accuracy; higher values indicate greater importance. Receiver operating characteristic (ROC) curves were plotted, and the area under the curve (AUC) was calculated to quantify model discrimination ability. Model discrimination was evaluated using receiver operating characteristic (ROC) curves on the testing set, and the area under the curve (AUC) was calculated.

### 2.6 Statistical analysis

Data analysis was conducted using R version 4.5.1 and SPSS version 26. Before analysis, variable-level missingness was assessed for the DED outcome and all candidate predictors. All variables had complete observations (*n* = 2,000; missing = 0, 0.0% for each variable). Accordingly, the conventional logistic regression was performed on the complete dataset, with no case excluded and no value imputed; the mixed-effects logistic regression and random forest analyses used the same dataset (*n* = 2,000). Continuous variables conforming to a normal distribution were expressed as mean ± standard deviation (SD) and compared using the *t*-test or analysis of variance (ANOVA). Non-normally distributed variables were expressed as median (interquartile range, IQR) and compared using the Mann–Whitney *U* test or Kruskal-Wallis *H* test. Categorical variables were expressed as counts (percentages) and compared using the chi-square (*χ*^2^) test.

Multicollinearity was assessed using dummy-variable VIFs. Region was excluded from the final model because its maximum VIF was 7.027 and it substantially overlapped with climate type and elevation, whose maximum VIFs were 6.884 and 6.315, respectively. Climate type and elevation were retained as more specific and interpretable environmental indicators. Factors associated with DED were initially evaluated using multivariable unconditional logistic regression, with ORs and 95% CIs reported. To quantify the effect of clustering on prevalence precision, a *post hoc* design-effect analysis was performed using the 40 subdistrict/township units as the analytical clusters. With an average cluster size of 50 and an ICC of 0.0173 estimated from the fitted random-intercept model, the design effect was 1.849 and the effective sample size was 1,081.7. A mixed-effects logistic regression model with the same fixed-effect specification as the conventional model and nested random intercepts for county/district and subdistrict/township clusters was additionally fitted as a sensitivity analysis. Random forest classification models were constructed using the “randomForest” package. The mean decrease in accuracy was used to assess the contribution of each predictor variable to model performance, with higher values indicating greater importance. ROC curves and AUC were used to evaluate model discrimination. Statistical significance was set at *p* < 0.05.

## 3 Results

### 3.1 Association of regional characteristics with DED

The survey revealed an overall DED prevalence of 27.5% in Gansu Province. The final sample comprised 2,000 participants recruited from the 40 sampled subdistrict/township clusters, corresponding to an average of 50 participants per cluster. The estimated ICC was 0.0173, yielding a design effect of 1.849 and an effective sample size of approximately 1,081.7. The standard error of the overall prevalence estimate increased from 0.0100 under the simple-random-sampling assumption to 0.0136 after design-effect adjustment, resulting in a design-adjusted 95% CI of 24.84–30.16%. Based on the anticipated prevalence of 30% and the prespecified absolute error of 2%, approximately 2,017 participants would have been required under simple random sampling and approximately 3,730 after incorporation of the observed design effect. Although the achieved sample size did not retain the prespecified absolute precision of 2%, it provided an actual absolute precision of approximately 2.66%, which remained within a 3% margin of error. Region-specific prevalence rates were 35.0% in the Hexi Corridor, 31.0% in Central Gansu, 23.3% in Southeastern Gansu, 32.5% in Gannan, and 15.8% in Longnan. Differences in prevalence among regions were statistically significant (*p* < 0.001), indicating substantial regional heterogeneity in DED prevalence. Based on elevation data from the Gansu Province Geographic Information Public Service Platform, participants’ residences were categorized into low elevation (<2,000 m), medium elevation (2,000–3,000 m), and high elevation (≥3,000 m) groups. DED prevalence was 23.0% in the low elevation group, 26.4% in the medium elevation group, and 38.4% in the high elevation group. Differences among the three groups were statistically significant (*p* < 0.001), and prevalence showed an increasing trend with elevation. According to climate type, DED prevalence was 43.6% in arid regions, 31.2% in semi-arid regions, 25.2% in semi-humid regions, and 16.8% in humid regions. Differences across climate types were statistically significant (*p* < 0.001), and prevalence increased with the dryness of the region (Table 2).

**Table 2:** Association of regional characteristics with DED prevalence.

Variables		Non-DED (*n* = 1,450)	DED (*n* = 550)	*χ* ^2^	*p*
Regions (*n*/%)	Longnan	337 (84.3%)	63 (15.8%)	50.082	<0.001
	Gannan	270 (67.5%)	130 (32.5%)		
	Southeastern Gansu	307 (76.8%)	93 (23.3%)		
	Central Gansu	276 (69.0%)	124 (31.0%)		
	Hexi corridor	260 (65.0%)	140 (35.0%)		
Climate type (*n*/%)	Humid	336 (83.2%)	68 (16.8%)	57.872	<0.001
	Semi-humid	535 (74.8%)	180 (25.2%)		
	Semi-arid	456 (68.8%)	207 (31.2%)		
	Arid	123 (56.4%)	95 (43.6%)		
Elevation (*n*/%)	<2,000 m	875 (77.0%)	262 (23.0%)	41.326	<0.001
	2,000–3,000 m	267 (73.6%)	96 (26.4%)		
	≥3,000 m	308 (61.6%)	192 (38.4%)		

### 3.2 Association of population characteristics and lifestyle with DED

Univariate analysis indicated that sex, age, daily screen time, work type, sleep quality, and smoking were significantly associated with DED (*p* < 0.05). In contrast, eyeglass use and alcohol consumption were not significantly associated with DED (*p* > 0.05; Table 3).

**Table 3:** Association of population characteristics and lifestyle factors with DED prevalence.

Variables		Non-DED (*n* = 1,450)	DED (*n* = 550)	*χ* ^2^	*p*
Sex (*n*/%)	Male	733 (74.6%)	250 (25.4%)	4.145	0.042
	Female	717 (70.5%)	300 (29.5%)		
Age (*n*/%)	18–35 years	576 (82.4%)	123 (17.6%)	72.687	<0.001
	36–55 years	524 (72.1%)	203 (27.9%)		
	>55 years	350 (61.0%)	224 (39.0%)		
Daily screen time (*n*/%)	≤6 h	778 (86.1%)	126 (13.9%)	152.105	<0.001
	>6 h	672 (61.3%)	424 (38.7%)		
Eyeglass use (*n*/%)	Without eyeglasses	813 (73.6%)	290 (26.4%)	1.610	0.204
	With eyeglasses	637 (71.1%)	260 (28.9%)		
Sleep quality (*n*/%)	PSQI <8	1,055 (75.7%)	338 (24.3%)	24.104	<0.001
	PSQI ≥8	395 (65.1%)	212 (34.9%)		
Work type (*n*/%)	Indoor work	613 (61.9%)	378 (38.1%)	111.608	<0.001
	Outdoor work	837 (83.0%)	172 (17.0%)		
Smoking status (*n*/%)	No	1,227 (76.8%)	371 (23.2%)	73.165	<0.001
	Yes	223 (55.5%)	179 (44.5%)		
Alcohol consumption (*n*/%)	No	1,090 (73.4%)	395 (26.6%)	2.346	0.126
	Yes	360 (69.9%)	155 (30.1%)		

### 3.3 Multivariable logistic regression analysis of DED risk factors

DED status was set as the dependent variable, and all significant variables selected on the basis of the univariable analysis and clinical relevance were entered into the multivariable logistic regression model. Multicollinearity diagnostics showed maximum dummy-variable VIFs of 7.027 for Region, 6.884 for climate type, and 6.315 for elevation, compared with values below 2.20 for the remaining predictors. Because Region represented a composite geographic classification that substantially overlapped with the more specific environmental indicators of climate type and elevation, it was excluded from the final model. The multivariable logistic regression model showed that climate type, elevation, sex, age, daily screen time, sleep quality, and smoking were independent associated with for DED (*p* < 0.05; Table 4). The overall model fit was further evaluated using the Hosmer-Lemeshow goodness-of-fit test and Nagelkerke *R*^2^. The Hosmer-Lemeshow test showed no evidence of poor calibration (*χ*^2^ = 13.987, *df* = 8, *p* = 0.082), indicating good agreement between predicted and observed probabilities. The Nagelkerke *R*^2^ was 0.236, suggesting that the included variables provided a modest explanatory capacity for DED occurrence.

**Table 4:** Multivariable logistic regression analysis of DED risk factors.

Variables	*B*	SE	*p*	OR	OR 95% CI	
					Lower limit	Upper limit
Female sex	0.389	0.120	0.001	1.475	1.165	1.868
Age 36–55 years	−0.082	0.151	0.588	0.921	0.685	1.239
Age >55 years	0.306	0.161	0.058	1.358	0.990	1.861
Semi-humid climate	0.777	0.189	<0.001	2.175	1.501	3.151
Semi-arid climate	1.713	0.241	<0.001	5.547	3.456	8.902
Arid climate	2.509	0.278	<0.001	12.295	7.130	21.200
Elevation 2,000–3,000 m	0.533	0.172	0.002	1.704	1.217	2.386
Elevation >3,000 m	1.888	0.197	<0.001	6.605	4.488	9.720
PSQI ≥8	0.487	0.135	<0.001	1.627	1.249	2.120
Daily screen time >6 h	0.985	0.163	<0.001	2.677	1.944	3.688
Outdoor work	0.093	0.165	0.573	1.097	0.794	1.516
Smoking	0.574	0.145	<0.001	1.776	1.337	2.360

Reference categories: male sex; age 18–35 years; humid climate; elevation <2,000 m; PSQI <8; daily screen time ≤6 h; indoor work; and non-smoking. OR, odds ratio; CI, confidence interval; PSQI, Pittsburgh sleep quality index; DED, dry eye disease.

### 3.4 Mixed-effects logistic regression sensitivity analysis

A mixed-effects logistic regression model was fitted as a sensitivity analysis to account for the hierarchical sampling structure. The fixed-effect specification was consistent with that of the final conventional logistic regression model, while nested random intercepts were included for county/district and subdistrict/township clusters. After adjustment for the included covariates and cluster-level random effects, daily screen time >6 h, age >55 years, smoking, arid climate, elevation of 2,000–3,000 m, elevation >3,000 m, poor sleep quality (PSQI ≥8) and female sex were associated with higher odds of DED, whereas no statistically significant associations were observed for outdoor work, age 36–55 years, semi-arid climate, or semi-humid climate (Table 5). The directions of the principal associations were generally consistent with the conventional model, although some age- and climate-specific estimates were attenuated after the clustering structure was incorporated. The estimate for elevation >3,000 m remained large and had a wide confidence interval and should therefore be interpreted cautiously.

**Table 5:** Mixed-effects logistic regression sensitivity analysis of factors associated with DED.

Variables	*B*	SE	*p*	OR	OR 95% CI	
					Lower limit	Upper limit
Female sex	0.377	0.124	0.002	1.458	1.143	1.858
Age 36–55 years	0.117	0.158	0.461	1.124	0.824	1.533
Age >55 years	0.539	0.171	0.002	1.713	1.226	2.394
Semi-humid climate	0.444	0.321	0.167	1.559	0.831	2.926
Semi-arid climate	0.392	0.211	0.063	1.480	0.979	2.238
Arid climate	0.995	0.405	0.014	2.706	1.222	5.991
Elevation 2,000–3,000 m	1.094	0.247	<0.001	2.986	1.838	4.851
Elevation >3,000 m	4.173	0.453	<0.001	64.942	26.744	157.695
PSQI ≥8	0.377	0.141	0.007	1.458	1.106	1.922
Daily screen time >6 h	0.789	0.174	<0.001	2.200	1.565	3.093
Outdoor work	0.196	0.175	0.262	1.217	0.864	1.714
Smoking	0.551	0.152	<0.001	1.736	1.287	2.340

Reference categories: male sex; age 18–35 years; humid climate; elevation <2,000 m; PSQI <8; daily screen time ≤6 h; indoor work; and non-smoking. OR, odds ratio; CI, confidence interval; PSQI, Pittsburgh sleep quality index; DED, dry eye disease.

### 3.5 Machine learning-based exploration of risk factors

A random forest model was constructed using regional characteristics, population characteristics, and lifestyle factors. Variable importance assessed by mean decrease in accuracy ranked climate type as the most important predictor of DED, followed by daily screen time and sleep quality, whereas elevation ranked fourth. ROC curve analysis showed an AUC of 0.832, indicating strong discriminative ability of the model. Accuracy was similar between the training set (82.8%) and testing set (78.2%), and out-of-bag (OOB) error (19.9%) closely matched testing error, demonstrating the model’s excellent generalizability. These results indicate that the random forest model performed robustly, remained stable, did not overfit, and exhibited strong predictive power (Figure 1).

**Figure 1:**
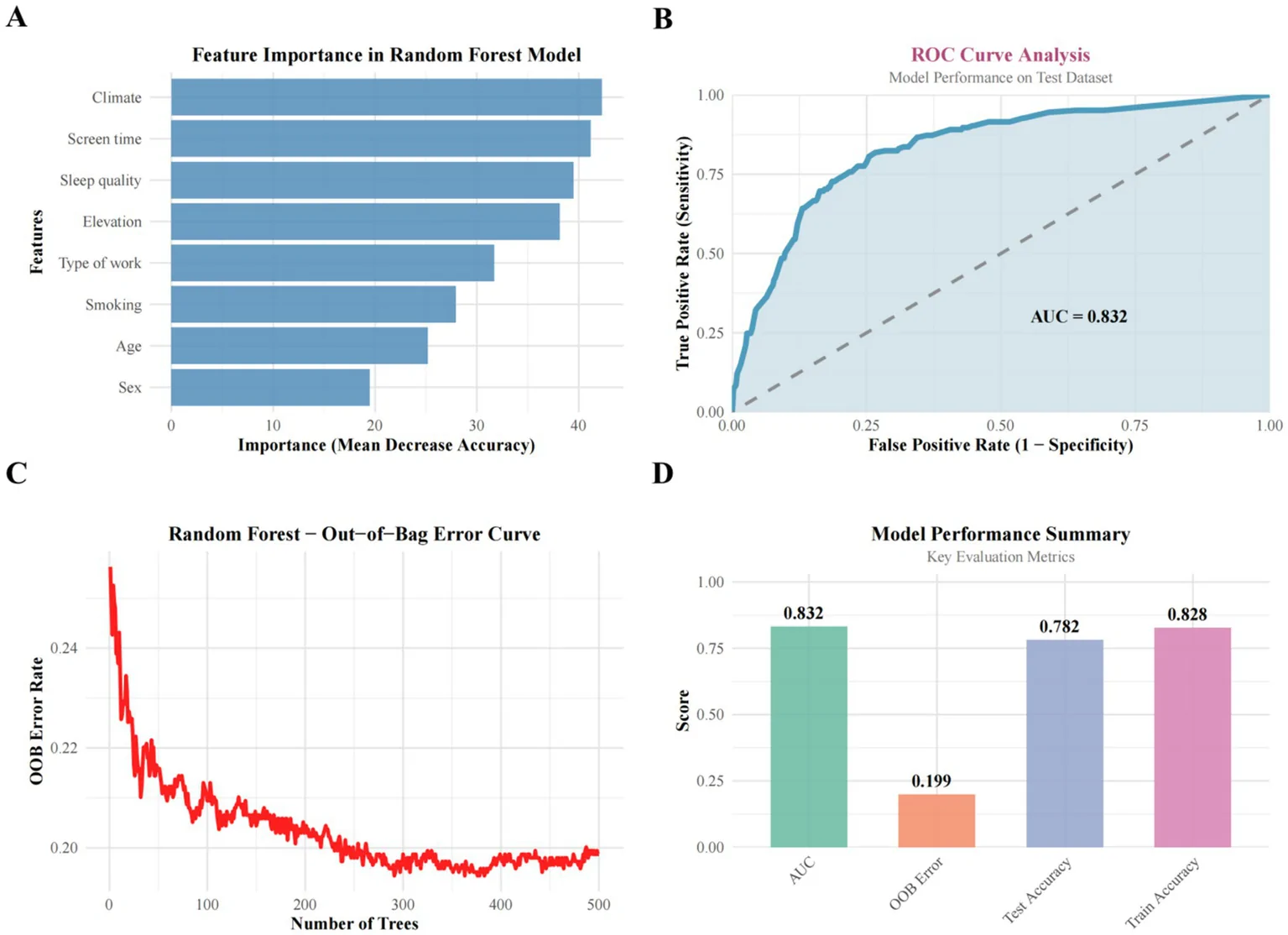
Comprehensive performance evaluation and feature analysis of the random forest model. **(A)** Variable importance ranking in the random forest model (based on mean decrease in accuracy). **(B)** ROC curve. **(C)** OOB error curve. **(D)** Summary of random forest model performance.

## 4 Discussion

In this study, we investigated the distribution and associated factors of DED across different geographic and climatic regions of Gansu Province using a combination of multivariable logistic regression and random forest analysis. The results demonstrated significant regional heterogeneity in DED prevalence, with climate type and elevation identified as the major environmental factors associated with DED. In addition, individual characteristics, including sex, daily screen time, sleep quality, and smoking, were independently associated with DED occurrence. Compared with previous studies that mainly focused on prevalence estimation or individual-level risk factors, this study further explored the contribution of geographic environmental gradients within an integrated analytical framework.

The geographic heterogeneity of Gansu provides a unique opportunity to explore the potential mechanisms linking environmental conditions with DED. The observed associations of climate type and elevation with DED suggest that macro-geographic environmental characteristics may play an important role in shaping ocular surface susceptibility. In the random forest analysis, climate type ranked first by mean decrease in accuracy, whereas elevation ranked fourth; this distinction indicates that both variables were independently associated with DED, although their relative contributions to model prediction differed. From northwest to southeast, Gansu spans the extremely arid Hexi Corridor, the temperate semi-arid Central Gansu, the transitional semi-humid to semi-arid Southeastern Gansu, the high-elevation humid Gannan, and the subtropical humid Longnan. This environmental gradient may influence ocular surface homeostasis through differences in tear evaporation, oxidative stress exposure, and inflammatory responses. In the extremely arid Hexi Corridor, low humidity combined with frequent sandstorms creates a high evaporative stress environment, accelerating tear evaporation and exposing the corneal epithelium to persistent physical stimuli, which disrupts epithelial integrity and triggers chronic inflammation (22, 23). High-elevation regions such as Gannan present a more complex “hypoxia–high radiation–low temperature” exposure pattern. Population-based studies involving older Tibetans and Mongolian residents in high-altitude areas of Qinghai have similarly documented a substantial burden of DED, with increasing altitude identified as an independent associated factor among older Tibetans. Together with previous findings from Hotan, Xinjiang (24, 25), these observations place the association identified in Gansu within a broader western Chinese context. Nevertheless, direct comparisons should be made cautiously because of differences in ethnicity, age distribution, diagnostic criteria, and accompanying climatic exposures across these populations. Chronic hypoxia may impair corneal limbal stem cell function, reducing ocular surface repair capacity (26). Intense ultraviolet (UV) radiation can induce excessive reactive oxygen species in ocular surface tissues, activate inflammatory signaling pathways, upregulate matrix metalloproteinase expression, degrade key components maintaining tear film stability, and promote meibomian gland lipid peroxidation, collectively disrupting tear film homeostasis (27). However, climate type and elevation should be interpreted as broader environmental indicators rather than isolated exposures, as these variables may represent the combined effects of multiple geographic and environmental characteristics. Beyond outdoor climatic conditions, indoor environmental exposures and behavioral adaptations may represent important intermediate factors linking regional climate to ocular surface responses. Occupational exposure may further influence this relationship. Although work type was included as an indoor/outdoor covariate, this binary classification did not capture the actual time spent outdoors or workplace-specific climatic conditions. Occupational exposure may therefore have produced residual confounding or partly mediated the association between residence-based climate type and DED; these pathways could not be distinguished in the present cross-sectional analysis. Recent evidence suggests that seasonal hygrothermal conditions and adaptive behaviors in indoor environments may influence dry eye symptoms by modifying ocular surface evaporative stress and comfort-related responses (28).

Our findings are generally consistent with previous epidemiological studies showing that demographic characteristics and lifestyle behaviors contribute to DED susceptibility. However, unlike previous investigations that primarily examined individual-level factors, our study further highlights the potential contribution of geographic environmental gradients when evaluated together with behavioral characteristics. At the individual level, risk factors do not exist in isolation but are interwoven with environmental exposures, jointly shaping clinical phenotypes. This study identified independent associations between climate type, elevation, sex, daily screen time, sleep quality, and smoking on DED risk. Higher prevalence among females is related to inherent effects of sex hormones on lacrimal secretion and local immune regulation (29). Long daily screen time was identified as one of the top three contributing factors, reflecting the profound impact of modern digital lifestyles on ocular health. Reduced blink frequency and incomplete blinking can lead to uneven tear distribution and accelerated evaporation, exacerbating visual fatigue and ocular surface inflammation (1, 30). Notably, sleep quality, as a systemic factor, may serve as a critical bridge linking psychological stress, the nervous system, and ocular surface health. Sleep deprivation can alter sympathetic and parasympathetic regulation of the lacrimal gland, disrupting secretion of nutritional and functional proteins, thereby reducing aqueous tear production (31). These mechanisms provide a possible biological explanation for the observed association between poor sleep quality and DED; however, the cross-sectional design of the present study does not establish the direction or underlying mechanisms of this relationship. From a public health perspective, these findings suggest that DED prevention strategies should consider both regional environmental characteristics and modifiable behavioral factors. Targeted health education, optimization of visual behavior, and environmental management may be valuable approaches for reducing DED burden in high-risk populations.

A methodological strength of this study was the complementary use of multivariable logistic regression and random forest models, enabling both effect estimation and assessment of variable importance. Logistic regression quantified the independent associations between candidate factors and DED, providing interpretable effect estimates. The random forest model further evaluated the relative contribution of different factors and captured potential nonlinear patterns among environmental and behavioral variables, thereby providing an additional perspective for understanding the complex risk structure of DED. The random forest model achieved an AUC of 0.832, indicating good discriminative ability based on combined environmental and behavioral characteristics. These findings suggest that machine learning approaches may provide complementary information for identifying high-risk populations and generating hypotheses for future validation studies.

Several limitations should be considered. First, the initial sample size calculation assumed simple random sampling and did not prospectively incorporate clustering. The *post hoc* design-effect analysis yielded an ICC of 0.0173 and a design effect of 1.849, reducing the effective sample size from 2,000 to approximately 1,081.7. The achieved sample size therefore did not retain the prespecified absolute precision of 2%, although it provided an actual precision of approximately 2.66%. Meanwhile, Because the numbers of residents initially approached or declining participation were not retained, overall and region-specific response rates could not be calculated. As participation required both a face-to-face questionnaire and ophthalmic examinations, potential selection and non-response bias should be considered when interpreting the prevalence estimates. Second, the mixed-effects sensitivity analysis accounted for the hierarchical sampling structure and yielded generally consistent directions for several principal associations; however, some age- and climate-specific estimates were attenuated, indicating that those findings were sensitive to cluster-level correlation. The estimate for elevation >3,000 m remained particularly large and imprecise. Because high elevation was concentrated in specific geographic areas, this estimate may partly reflect residual geographic heterogeneity, sparse covariate combinations, and overlap between elevation and climate rather than the isolated effect of elevation itself. Third, DED was analyzed as a single binary outcome because the test-specific grades could not be combined using a prespecified validated overall severity algorithm. Although this approach maintained consistency with the primary case definition, it may have obscured differences in risk-factor profiles between mild and severe disease. Future studies should apply a validated composite severity-classification system to examine severity-specific associations. Fourth, although core environmental and behavioral variables were included, the binary indoor/outdoor work classification did not capture the actual duration of outdoor exposure, workplace location, or workplace microclimate, while indoor humidity and heating or cooling conditions were not measured. Residual confounding or mediation by occupational and indoor environmental exposure therefore cannot be excluded when interpreting the residence-based climate associations. In addition, finer pathophysiological mediators, such as meibomian gland morphology and function, or tear inflammatory cytokine profiles, were not analyzed, limiting direct mechanistic interpretation. Future prospective studies should incorporate the design effect during sample-size planning, retain complete sampling-unit identifiers, and combine multilevel analyses with detailed environmental and biological measurements.

In conclusion, this study used mathematical modeling to construct a comprehensive analytic framework spanning macro-ecology to individual behavior, enriching regional DED epidemiological data in China and providing critical theoretical and practical guidance for implementing scientific and precise DED prevention and management strategies in Gansu.

## Data Availability

The original contributions presented in the study are included in the article/supplementary material, further inquiries can be directed to the corresponding author.
